# Activating transcription factor 3 promotes embryo attachment via up-regulation of leukemia inhibitory factor in vitro

**DOI:** 10.1186/s12958-017-0260-7

**Published:** 2017-06-02

**Authors:** Xi Cheng, Jingyu Liu, Huizhi Shan, Lihua Sun, Chenyang Huang, Qiang Yan, Ruiwei Jiang, Lijun Ding, Yue Jiang, Jianjun Zhou, Guijun Yan, Haixiang Sun

**Affiliations:** 1 Reproductive Medicine Center, The Affiliated Drum Tower Hospital of Nanjing University Medical School, Nanjing, 210008 People’s Republic of China; 2 Reproductive Medicine Center, Shanghai Ninth People’s Hospital Affiliated Shanghai JiaTong University School of Medicine, Shanghai, 200011 People’s Republic of China; 3 Collaborative Innovation Platform for Reproductive Biology and Technology of Nanjing University Medical School, Nanjing, 210008 People’s Republic of China; 4 Center of Reproductive Medicine, Nanjing Jinling Hospital, the Medical School of Nanjing University, Nanjing, 210002 China

**Keywords:** ATF3, LIF, Embryo adhesion, Recurrent implantation failure

## Abstract

**Background:**

A receptive endometrium is essential for maternal-embryonic molecular communication during implantation. However, the specific molecular regulatory mechanisms of the endometrial capacity remain poorly understood. Here, we examined activating transcription factor 3 (ATF3) expression in human endometria and the functional effect of ATF3 on embryo attachment in vitro.

**Methods:**

Immunohistochemistry (IHC) was used to assess the ATF3 expression patterns in human endometria. Quantitative real-time PCR (qRT-PCR), western blotting and immunofluorescence (IF) studies were applied to explore ATF3 expression in Ishikawa cells upon estrogen (E_2_) and medroxyprogesterone acetate (MPA) treatment. qRT-PCR and western blotting were performed to inspect LIF (leukemia inhibitory factor) expression after enhancement or inhibition of ATF3, and a luciferase reporter assay and ChIP-PCR were used to confirm the regulatory mechanism of ATF3 to LIF. Endometrial epithelial capacity was assessed by an in vitro model of attachment of BeWo spheroids to Ishikawa cells. Western blotting was performed to compare the expression of ATF3 in endometrial samples of recurrent implantation failure (RIF) patients with that in samples from fertile women (FER) who had undergone no less than one successful embryo transplantation.

**Results:**

ATF3 was located in human endometrial epithelial cells and stromal cells and was significantly induced by E_2_ and MPA in Ishikawa cells. Adenovirus-mediated overexpression of ATF3 in Ishikawa cells activated LIF promoter activity and enhanced its expression. Accordingly, the stimulation of BeWo spheroid adhesion promoted by ATF3 was inhibited by pretreatment with a specific antibody against LIF via the antibody-blocking assay. Moreover, ATF3 was aberrantly decreased in the endometria of RIF patients.

**Conclusions:**

Our findings suggest that ATF3 plays a significant role in regulating human endometrial receptivity and embryo attachment in vitro via up-regulation of leukemia inhibitory factor.

**Trial registration:**

Construction and management of the Nanjing multi-center biobank. No. 2013-081-01. Registered 10 Dec. 2013.

**Electronic supplementary material:**

The online version of this article (doi:10.1186/s12958-017-0260-7) contains supplementary material, which is available to authorized users.

## Background

Implantation is a dynamic and highly coordinated event that involves the appropriately timed attachment of the viable blastocyst and its invasion into the receptive endometrium [1]. The human endometrium is repeatedly remodeled during the sexual cycle and is prepared for implantation by the synergistic effects of steroid hormones, adhesion molecules, growth factors and extracellular matrix proteins [2]. Impaired or improper endometrial receptivity is currently considered as the most important limiting factor for the establishment of pregnancy, particularly regarding implantation failure of high-quality embryos during assisted reproduction [3].

Several different signaling pathways and associated genes have been reported to participate in the adjustment of human endometrial activity, such as homeobox-containing transcription factor 10 (Hoxa10) [4], signal transducer and activator of transcription 3 (STAT3) [5], and leukemia inhibitory factor (LIF) [6]. Most of the signaling pathways involve ovarian steroid hormones [7]. LIF, a pleiotropic cytokine secreted by glands, directly targets the epithelium and triggers the increased expression of several proteins (HB-EGF, cochlin, IGFBP-3, and IRG1) that promote uterine receptivity [8]. Along with signals from the blastocyst, LIF is required for the initiation of decidualization by activating STAT3 [9]. Concentrations of LIF in uterine flushing obtained from infertile women have been found to be markedly low compared to fertile controls, indicating that LIF dysregulation may be responsible for decreased uterine availability and implantation failures, such as recurrent implanting failure or abortion [10]. Although LIF is essential in embryo-endometrial interactions, there is limited information available regarding the molecular regulation of LIF expression.

Activating transcription factor 3 (ATF3) is a member of the ATF/cAMP response element binding protein (CREB) family of transcription factors and contains a basic region and a leucine zipper [11]. ATF3 is highly induced in response to multiple extracellular signals and is involved in cell cycling [12], neutrophil migration [13] and sexual differentiation [14]. ATF3 was reported to be responsive to estrogen [15], progestogen [16] and prostaglandin [17] in specific cells in different physiological or pathological conditions.

In this study, ATF3 is shown to be a novel downstream target gene induced by estrogen (17β-estradiol, E_2_) and medroxyprogesterone acetate (MPA) in Ishikawa cells. We demonstrate that ATF3 specifically promotes LIF expression and facilitates embryo attachment. These results establish that ATF3 regulates human endometrial receptivity and offer a new molecular mechanism to consider in studies of the capacity of endometrial epithelial cells during implantation.

## Methods

### Patient samples

The patients enrolled in this study were recruited from the in vitro fertilization (IVF) unit of the Reproductive Center of the Affiliated Drum Tower Hospital of Nanjing University Medical School. All of the participants were 22 - 38 years old, with a body mass index (BMI) of 18 - 26 kg/m^2^ and regular menses. Among the participants from which secretory phase endometria were obtained, the endometrium thickness was 8 - 14 mm. A total of 15 recurrent implantation failure (RIF) patients, who had failed to achieve pregnancy after at least four good-quality embryo transfers over a minimum of three fresh or frozen cycles, were enrolled. Thirteen age-matched fertile controls with once embryo transplantation were also enrolled (Table 1). Secretory phase endometria were obtained from the RIF patients (*n* = 15) and fertile controls (*n* = 13) within days 19 to 23 of menstruation by diagnostic uterine curettage (Additional file 1: Table S1). A total of 10 proliferative phase endometria were obtained from patients undergoing endometrial biopsy within day 5 to day 12 of menstruation with the single diagnosis of fallopian tube jam, excluding endometrial disease such as endometriosis, uterine fibroid, intrauterine adhesion, and endometrial hyperplasia. Ten secretory phase endometrial tissues were obtained from age-matched fertile controls within days 19 to 23 of menstruation via diagnostic uterine curettage (Table 2).

**Table 1:** Demographic details of the fertile control (FER) and RIF patient participants in the study of endometrial ATF3 expression

Fertility status	FER (*n* = 13)	RIF (*n* = 15)	*p*
Age (years)	32.5 ± 4.12	33.2 ± 4.44	0.6877
BMI (kg/m^2^)	20.7 ± 2.11	21.5 ± 2.28	0.385
Endometrial thickness (mm)	9.1 ± 1.50	10.6 ± 2.24	0.125
No. of transferred embryos	2 ± 0	8.267 ± 3.83	1.0533E-06

The data are presented as the mean ± SD. *p* < 0.05 was considered significant

**Table 2:** Demographic details of the participants in the study of endometrial ATF3 expression according to endometrial phase

Phase of menstruation	Proliferative phase (*n* = 10)	Secretory phase (*n* = 10)	*p*
Age (years)	32.0 ± 3.80	34.6 ± 3.03	0.108
BMI (kg/m^2^)	20.7 ± 1.185	21.9 ± 2.677	0.226

The data are presented as the mean ± SD. *p* < 0.05 was considered significant

This study was approved by the institutional review board of the Affiliated Drum Tower Hospital of Nanjing University Medical School on December 5, 2013 (2013–081–01), and signed informed consent was obtained from all patients.

### Cell culture

The Ishikawa cells and BeWo cells used in this study were donated by Dr. Yali Hu from Department of Gynecology and Obstetrics in the Affiliated Drum Tower Hospital of Nanjing University Medical School. The cells were cultured in Dulbecco’s modified minimum essential medium (HyClone, Thermo Scientific, South Logan, UT, USA) containing 10% fetal bovine serum (FBS, Gibco BRL/Invitrogen, Carlsbad, CA, USA) and 1% penicillin/streptomycin (HyClone). To simulate physiological conditions, the Ishikawa cells were cultured at the concentration of 1 × 10^4^ cells/cm^2^, and treated or not treated with 10 nM E_2_ and 1 μM MPA (Sigma, St. Louis, MO, USA) for 0.5, 1, 2, 3, 4, 6 and 9 h with 2% charcoal/dextran-treated FBS and 1% penicillin/streptomycin in DMEM/F12 at 37 °C. ICI182780 (V900926, Sigma) and mifepristone (M8046, Sigma) were used at a concentration of 1 μM for 24 h to block estrogen receptors (ESR) and progesterone receptors (PR) in Ishikawa cells.

Human endometrial stromal cells (hESCs) were isolated using standard procedures [18]. Secretory endometrial tissues were minced and enzymatically digested with 0.1% collagenase (Worthington, Freehold, NJ, USA) for 30 min at 37 °C. Stromal cells were separated from intact glands by filtration of the digested tissue through 40 μm gauze. After centrifugation at 800 g for 5 min, the pellet was resuspended in DMEM/F12 (Gibco BRL/Invitrogen, Carlsbad, CA, USA) containing 10% charcoal/dextran-treated FBS (HyClone; Thermo Scientific, South Logan, UT, USA). The endometrial stromal cells were then maintained in DMEM/F12 supplemented with 10% charcoal/dextran-treated FBS and 1% penicillin/streptomycin at 37 °C. The cultured stromal cells were 95% pure, as judged by vimentin staining [19].

Primary cultures of human endometrial epithelial cells (hEECs) were prepared as described previously [20]. Secretory endometrial tissues were minced and enzymatically digested with 0.1% collagenase (Worthington, Freehold, NJ, USA) for 30 min at 37 °C. After incubation at above condition, the cell suspension was passed through 40 μm gauze to separate the single cells from undigested tissue. The unstrained tissues were resuspended in DMEM/F12 containing 10% charcoal/dextran-treated FBS and 1% penicillin/streptomycin and were transferred to dishes for 30 min to allow the adherence of contaminating stromal cells. Non-adherent cells/glands were filtered through 40 μm gauze 3 times to wash off the single cells, and the non-filtered pellets were resuspended and cultured. The endometrial epithelial cells were then maintained in DMEM/F12 supplemented with 10% charcoal/dextran-treated FBS and 1% penicillin/streptomycin at 37 °C. The cultured stromal cells were 90% pure, as judged by cytokeratin staining [19].

### RNA isolation and quantitative real-time PCR (qRT-PCR)

Total RNA was extracted from Ishikawa cells using TRIzol reagent (Invitrogen) according to the manufacturer’s instructions. A 1 μg aliquot of purified total RNA was reverse transcribed into cDNA using a Prime Script RT reagent kit (Takara-Bio, Otsu, Japan). qRT-PCR was performed using a SYBR green kit (Bio-Rad Laboratories, Hercules, CA, USA). The specific primers were as follows: human ATF3, 5′- TCGGAGAAGCTGGAAAGTGT-3′ and 5′-TCTGGAGTCCTCCCATTCTG-3′; human LIF, 5′-AGTCGTGACCTTGGCACCTC-3′ and 5′-GTTGACAGCCCAGCTTCTTC-3′; and human 18S rRNA, 5′-CGGCTACCACATCCAAGGAA-3′ and 5′-CTGGAATTACCGCGGCT-3′. The reactions were performed using a MyiQ Single-Color Real-time PCR Detection System (Bio-Rad). The PCR conditions were as follows: 95 °C for 15 min, followed by 40 cycles of 95 °C for 15 s and 60 °C for 30 s. The fold change in the expression of each gene was normalized to the expression of the endogenous control (18S rRNA), and the data were analyzed using the 2^-ΔΔCT^ method.

### Western blotting

Protein extracts were prepared from endometria or cells that were washed with ice-cold phosphate-buffered saline (PBS) and lysed in whole cell lysis buffer (50 mM Tris–HCl [pH 7.6], 150 mM NaCl and 1.0% NP-40) containing a protease inhibitor cocktail and phosphatase inhibitor cocktails 2 and 3 (Sigma). Equal amounts of total protein (30 μg) were separated on 12% (w/v) SDS-polyacrylamide gel and transferred to PVDF membranes (Millipore, Billerica, MA, USA). The membranes were blocked in Tris-buffered saline solution containing 5% nonfat milk for 1 h and then exposed to the following primary antibodies: ATF3 (1:1000; HPA001562, Sigma, 22 kD), LIF (1:500; L0669, Sigma; 38 kD), STAT3 (1:1000; #12640, Cell Signaling Technology, Danvers, MA, USA, 86 kD), p-STAT3 (1:1000; #9145, Cell Signaling Technology, 90 ~ kD), His-Tag antibody (1:2000; M30111, Abmart, Shanghai, China), and GAPDH (1:10000; AP0063, Bioworld, Nanjing, China; 42 kD). Immunodetection was accomplished using goat anti-rabbit or donkey anti-mouse secondary antibodies and an enhanced chemiluminescence detection kit (Millipore). The density of each band was scanned and measured by ImageJ software with target protein that had been normalized to GAPDH.

### Immunohistochemical staining

After the endometrial samples were dewaxed, endogenous peroxidase activity was blocked using freshly prepared PBS containing 0.3% hydrogen peroxide for 15 min. Antigen retrieval was conducted by autoclaving the samples at 121 °C for 15 min in the presence of EDTA (pH = 9.0). The sections were washed with PBS and then incubated with antibodies against ATF3 (1:1000; HPA001562, Sigma) overnight at 4 °C in a humidified chamber. The sections were subsequently rinsed with PBS and incubated with a horseradish peroxidase (HRP)-conjugated goat anti-rabbit secondary antibody at 37 °C for 30 min. HRP activity was detected using diaminobenzidine (Invitrogen), and the sections were counterstained with hematoxylin. Nonspecific rabbit serum (Boster, Wuhan, China) was used as a negative control.

### Immunofluorescence staining

Ishikawa cells plated on 18 mm micro-cover glasses were subjected to E_2_ and MPA for 6 h. Treated or untreated Ishikawa cells were fixed with 4% paraformaldehyde in PBS for 30 min at room temperature. After washing with PBS, the cells were permeabilized with 0.2% Triton X-100 in PBS for 15 min at room temperature. After blocking with 1% bovine serum albumin (BSA) in PBS, the cells were probed for ATF3 and F-actin and then incubated at 4 °C overnight with anti-ATF3 polyclonal antibody (1:1000; HPA001562, Sigma) and F-actin (1:300, P5282, Sigma), with or without Alexa Fluor 594-conjugated goat anti-rabbit IgG (1:200; Invitrogen). Nuclei were stained with 4′,6-diamidine-2-phenylindole (DAPI), which was included in the Vectashield Mounting Medium for Fluorescence with DAPI kit. Images were visualized using a confocal microscope (Leica).

### Chromatin immunoprecipitation (ChIP)/PCR assay

Ishikawa cells (70% confluence) were infected with Ad- ATF3-His (abm, Canada) or Ad-LacZ (Clontech, Japan) for 48 h. Cells were then washed with PBS and crosslinked with 1% formaldehyde for 15 min at room temperature. Crosslinking was stopped with the addition of glycine (0.125 M final concentration) for 10 min. Cells were washed twice with cold PBS, harvested in lysis buffer (20 mM Tris–HCl [pH 8.0], 85 mM KCl, 1 mM EDTA, 0.5 mM EGTA, 0.5% Nonidet P40, and protease inhibitor cocktail [Sigma]), and pelleted by centrifugation. Cell pellets were then lysed in nuclear lysis buffer (50 mM Tris–HCl [pH 8.0], 10 mM EDTA, 1% SDS, and protease inhibitor cocktail) and sonicated on ice to yield genomic DNA fragments with sizes of approximately 500–2000 base pairs. Next, precleared sonicates were immunoprecipitated using ATF3 antibody (HPA001562, Sigma) and nonspecific IgG as a technical control. Beads were collected and washed extensively. Immune complexes were eluted by incubation with fresh elution buffer (1% SDS and 0.1 M NaHCO3) at 65 °C for 30 min and then incubated at room temperature for 15 min. Crosslinks were reversed by incubation with NaCl at a final concentration of 0.3 M for 5 h at 65 °C. The eluates were incubated with proteinase K, and DNA was purified by phenol-chloroform extraction and ethanol precipitation. Finally, the purified DNA fragments were used as a template for PCR amplification. The specific primers that were used to amplify the LIF promoter DNA fragments containing an ATF3 binding sequence were 5′-GGCCTAGTAACCTCTGCTC-3′ and 5′-CGGCTCCCTCATGGAAG-3′ (spanning 232 bp;−2756 to−2497) as well as 5′-GCGAGGCTGCAAGAGCTC-3′ and 5′-CATGCCGTCCCTAAAGCTGC-3′ (spanning 150 bp; −1331 to−1181).

### Transient transfection and luciferase reporter assay

The wild-type human LIF promoter sequence (−2756 to−2497,−1331 to−1181,−1023 to 0) was amplified by PCR using Ishikawa cell genomic DNA with the primers 5′-GCATGGTACCGGCCTAGTAACCTCTGCTC-3′ and 5′-GCGCCTCGAGCGGCTCCCTCATGGAAG-3′, 5′-GCATGGTACCGCGAGGCTGCAAGAGCTC-3′ and 5′-GCGCCTCGAGCATGCCGTCCCTAAAGCTGC-3′ and 5′-GCATGGTACCTGGAGGTGTCCCTGTGCTC-3′ and 5′-GCGCCTCGAGGGATCCCCAGTCCAGGAAG-3′. PCR product was cloned into the pGL3-basic luciferase reporter plasmid (Promega). Preconfluent (60%) Ishikawa cells were transfected with the indicated plasmids using Lipofectamine 2000 (Invitrogen). The cells were incubated for an additional 48 h and then harvested for preparation of cell extracts. Luciferase activity was measured with a Luciferase Assay System (Promega, Madison, USA) in which Renilla luciferase plasmids were cotransfected as controls to standardize the transcription efficiency. These assays were performed using a Centro XS3 LB 960 luminometer (Berthold Technologies, BW, Germany) according to the manufacturer’s instructions.

### Assay for the attachment of BeWo spheroids to Ishikawa cells

According to our standard laboratory protocol [21], we used multicellular spheroids of human choriocarcinoma BeWo cells with endometrial Ishikawa cells as an in vitro model of attachment. BeWo cells were detached with 0.25% trypsin (Gibco) after reaching 80% confluence. The BeWo cell suspensions were then placed in 35 mm^2^ dishes coated with an anti-adhesive polymer, poly-2-hydroxyethyl methacrylate (polyHEMA, Sigma), to induce the formation of BeWo spheroids that were 150–200 μm in diameter after 48 h of culture. Simultaneously, the confluent monolayer Ishikawa cells grown to 50% confluency in 24-well plates were infected with Ad-ATF3-His and Ad-LacZ (at a multiplicity of infection (MOI) of 0 or 40) for 48 h or were transfected with si-ATF3 and si-CTL (0, 50, 100 nM) for 48 h. A functional blocking antibody against LIF was also used in the attachment assay as the adenovirus-treated cells were cultured in the presence of a mouse monoclonal antibody against LIF (L0669, Sigma) or the mouse preimmune IgG antibody (Abcam, Cambridge, MA, USA) at a concentration of 0.5 μg/mL for 1 h (Additional file 2: Figure S1) before the transfer of the BeWo spheroids onto the surface of a confluent monolayer of Ishikawa cells. After incubation at 37 °C for 2 h, any unattached spheroids were removed by washing the cells with PBS containing Ca^2+^ (0.1 mg/L) and Mg^2+^ (0.1 mg/L). Then, attached spheroids were counted under a light microscope, and the attachment rate was expressed as the percentage of the total number of spheroids added to the Ishikawa monolayer. All of the attachment assays were performed in triplicate.

### Statistical analysis

Unless stated otherwise, the numerical data are presented as the mean ± SD of at least three independent experiments. Student’s *t*-test was used to test for differences between two groups. ANOVA with Bonferroni correction was employed for multiple comparisons. Pearson correlation analysis was used to assess the relationship between ATF3 and LIF. Differences were considered significant at *p* < 0.05.

## Results

### ATF3 is expressed in the human endometrium

**Fig. 1:**
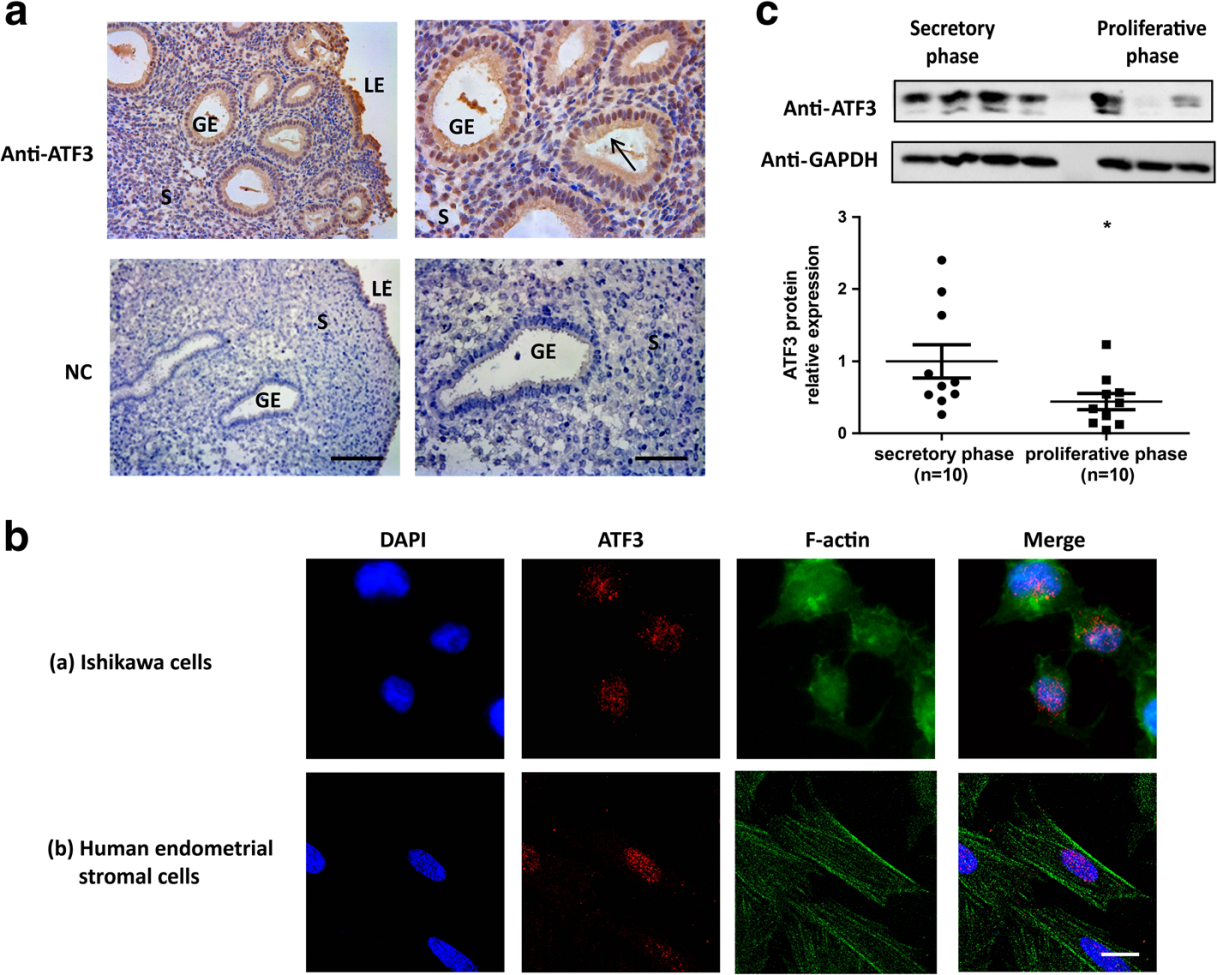
Expression of ATF3 in human endometrium. (**A**) Immunohistochemical analysis with the ATF3 antibody. Secretory endometrial tissue samples from fertile women are shown at 200× *(left panel)* and 400× *(right panel)* magnification. The negative control (NC) is nonspecific rabbit serum. Brown staining represents positive staining (arrows). Scale bars, 100 μm *(left panel)* and 50 μm *(right panel)*. (**B**) Immunofluorescence images of endogenous ATF3 and F-actin in Ishikawa cells (a) and human endometrial stromal cells (b). Scale bars, 25 μm. (**C**) ATF3 protein expression in secretory phase (*n* = 10) and proliferative phase endometria (*n* = 10). *p < 0.05 vs. the control group

Secretory phase endometria from normal cycling women who had not been treated with hormones in the last three months were used to examine ATF3 expression in the human endometrium. As shown in Fig. 1A, we observed specific staining for the ATF3 protein in the human endometrium, both in the epithelial cells and stromal cells, compared with that of the negative control. Endogenous ATF3 was mainly localized in the nucleus in Ishikawa cells (Fig. 1B-a) as well as human endometrial stromal cells (Fig. 1B-b), as observed with immunofluorescence staining. ATF3 expression was greater in the secretive phase (*n* = 10) than in the proliferative phase of the endometria (*n* = 10) (*p < 0.05, Fig. 1C, Additional file 1: Table S2).

### ATF3 is up-regulated by E_2_ and MPA in Ishikawa cells

**Fig. 2:**
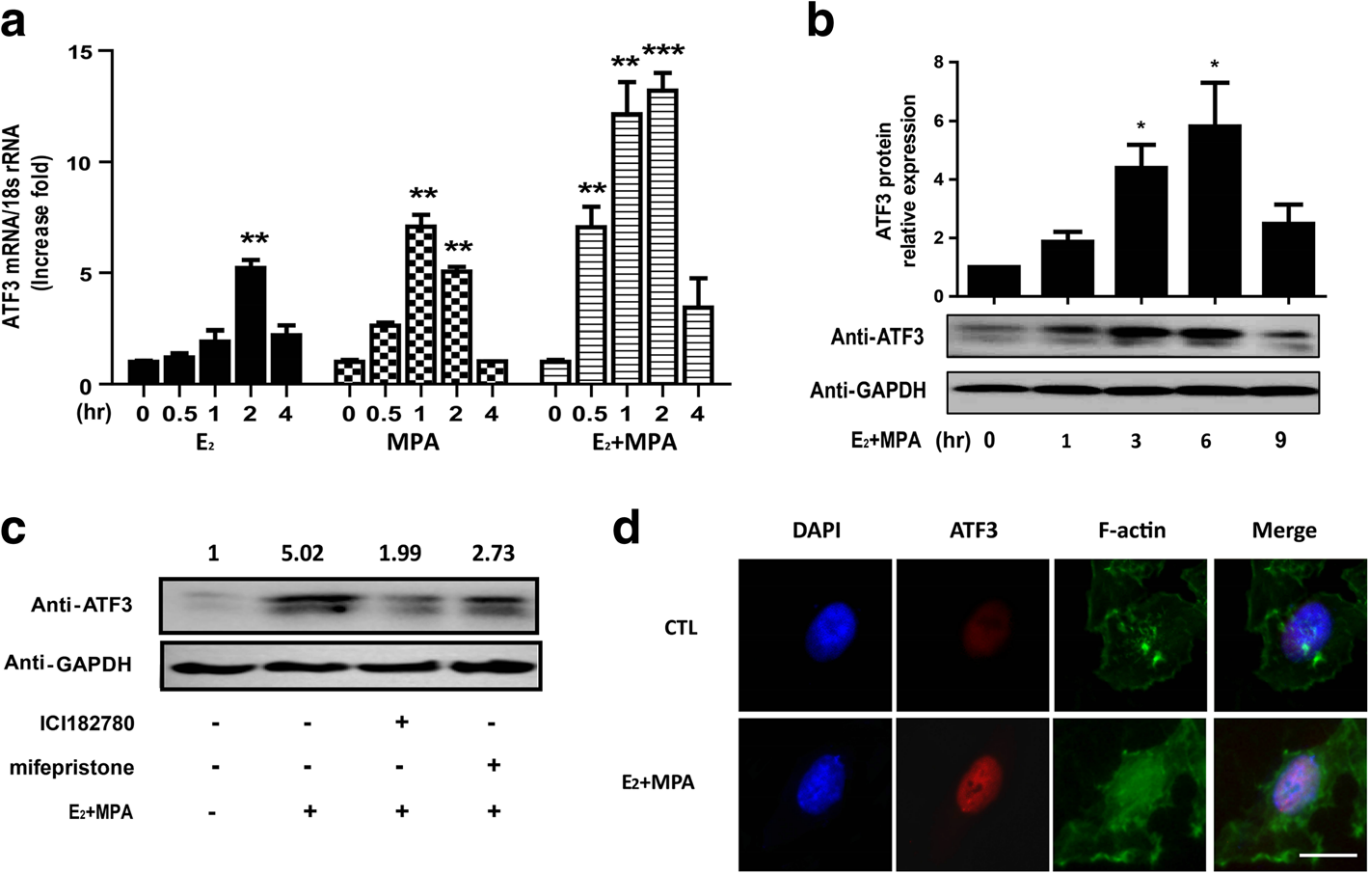
Up-regulation of ATF3 in Ishikawa cells upon treatment with E_2_ and MPA. Ishikawa cells were treated with E_2_, MPA, or both E_2_ and MPA for the indicated time periods. ATF3 mRNA levels (**a**) were measured by real-time PCR. ATF3 protein expression (**b**) was determined by Western blot and was quantitated by densitometric analysis. *p < 0.05; **p < 0.01; ***p < 0.001 compared with untreated controls. **c** Pretreatment of ICI182780 and mifepristone was performed before sex hormones were analyzed. ATF3 protein expression was determined by Western blot. **d** Immunofluorescence images demonstrated endogenous ATF3 expression and F-actin demonstration in Ishikawa cells treated with E_2_ and MPA for 6 h. Scale bars, 25 μm

ATF3 mRNA expression was enhanced five-fold after 2 h by either E_2_ or MPA in a time-dependent manner in Ishikawa cells (Fig. 2a). When stimulated with both E_2_ and MPA, the ATF3 mRNA level in Ishikawa cells had increased 7-fold at 0.5 h and over 10-fold at 2 h (Fig. 2a). ATF3 protein expression began to increase from 1 h and reached a maximum (approximately 5-fold) increase at 6 h (*p < 0.05, Fig. 2b). ATF3 protein expression decreased following exposure to pretreatment with ICI182780 and mifepristone (Fig. 2c). We also determined that ATF3 mRNA level was declined with pre-treatment of ICI182780 and mifepristone were performed before sex hormones were perfomed (Additional file 2: Figure S2).  Immunofluorescence staining demonstrated that ATF3 accumulated predominantly in the nucleus upon E_2_ and MPA treatment (Fig. 2d).

### ATF3 facilitates embryo attachment in vitro

**Fig. 3:**
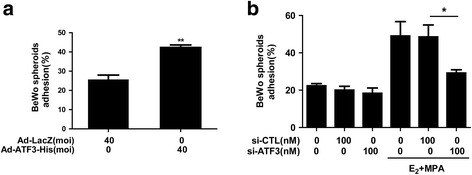
ATF3 facilitates embryo attachment in vitro. **a** Ishikawa cells were transduced with Ad-LacZ and Ad-ATF3-His at 0 or 40 MOI for 48 h. **b** Ishikawa cells were exposed to E_2_ and MPA for 24 h after 48 h transfection with 0 or 100 nM si-CTL or si-ATF3. Adhesion experiments were performed to assess BeWo spheroid attachment to the Ishikawa cell monolayer. The data represent the results of 3 independent experiments. ANOVA was used to compare the percentage of the attached spheroids in each treatment with that of the control. **p < 0.01 vs. the Ad-LacZ group. *p < 0.05 vs. the si-CTL group

As shown in Fig. 3a, overexpression of ATF3 in Ishikawa cells increased the ratio of BeWo spheroid adhesion by 1.6-fold compared with that of the Ad-LacZ group (25.6 ± 4.19% vs. 42.3 ± 2.33%, **p < 0.01). In contrast, siRNA-mediated knockdown of endogenous ATF3 protein expression in Ishikawa cells reversed the facilitating effect of E_2_ and MPA on BeWo spheroid attachment: 48.5 ± 15.78% (si-CTL) vs. 29.2 ± 4.31% (si-ATF3) (*p < 0.05, Fig. 3b). These data suggest that ATF3 contributes to embryo attachment in vitro.

### ATF3 transcriptionally increases LIF expression

**Fig. 4:**
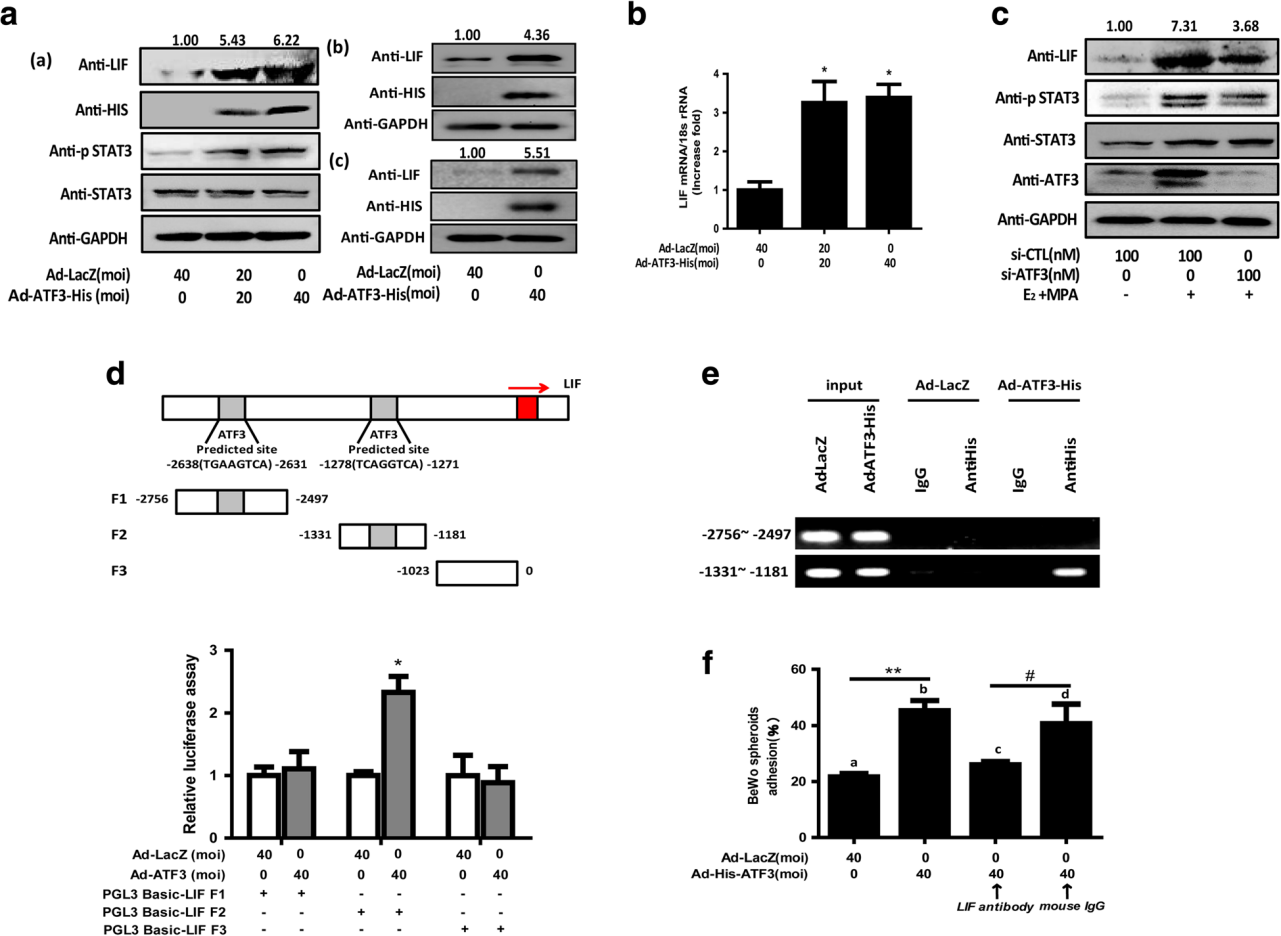
ATF3 transcriptionally increases LIF expression. (**A**, **B**) Ishikawa cells (a), primary endometrial epithelial cells (b) and stromal cells (c) were transduced with Ad-LacZ and Ad-ATF3-His at 0, 20 and 40 MOI for 48 h. (**C**) Ishikawa cells were exposed to E_2_ and MPA for 24 h after 48 h transfection with 0, 50, or 100 nM si-CTL or si-ATF3. LIF, STAT3, p-STAT3, HIS and GAPDH protein levels were measured by Western blotting assays. LIF mRNA expression levels were measured by qRT-PCR. *p < 0.05 vs. the Ad-LacZ group. The error bars indicate ± SD of 3 independent experiments. The density of LIF protein was analyzed and is presented. (**D**) Ishikawa cells were transfected with 500 ng firefly luciferase reporter plasmids (LIF-Luc) after transfecting Ad-His-ATF3 or Ad-LacZ for 24 h. Luciferase assays were performed, and the resulting data were normalized to constitutive *Renilla* luciferase levels (*n* = 3). (*p < 0.05). (**E**) Co-precipitated chromatin was amplified by PCR using primers specific for the LIF promoter region. PCR products were separated by agarose gel electrophoresis. Input (non-precipitated) chromatin was used as a positive control for these analyses. (**F**) Antibodies against LIF or mouse preimmune IgG were cultured with treated cells at a concentration of 0.5 μg/mL for 1 h before the transfer of the BeWo spheroids. **p < 0.01 Ad-ATF3 (b) vs. Ad-LacZ (a); ^#^ *p* < 0.05 anti-ATF3 (d) vs. IgG control (d). The error bars indicate ± SD of 3 independent experiments

Next, we discovered that ATF3 distinctly promoted the expression of LIF, consequently activating the phosphorylation of the STAT3 pathway (Fig. 4A-a, B) in Ishikawa cells. Up-regulation of LIF expression induced by ATF3 was also found in primary human epithelial cells (Fig. 4A-b) and stromal cells (Fig. 4A-c). In contrast, hormone-induced LIF expression was significantly reduced by knocking down ATF3 in Ishikawa cells (Fig. 4C).

ATF3 binds to a consensus DNA sequence (TGACGTCA) and forms selective heterodimers via the leucine zipper region [22]. We visually scanned the wild-type LIF (–3500 to +65) and found the specific ATF3 binding sites in the promoters of LIF (–2756 to–2497,–1331 to–1181). We generated 3 LIF-Luc reporter constructs, namely F1-Luc, F2-Luc, and F3-Luc (Fig. 4D). The reporter gene assay revealed a marked increase in luciferase activity of the fragment of−1331 to–1181 but no change in the fragments coding−2756 to–2497 and−1023 to 0. Conventional ChIP-PCR analysis was used to investigate whether the LIF promoter is a direct target for ATF3 in Ishikawa cells. As shown in Fig. 4E, the F2 promoter region (−1331 to−1181) was effectively recovered from immunoprecipitates of ATF3 proteins, but it was not recovered from those of the LacZ control and F1 promoter region (−2756 to−2497) of the LIF gene.

An antibody-blocking assay further demonstrated that the ATF3-mediated promotion of BeWo spheroid adhesion to Ishikawa cells [attachment rate: 45.3 ± 6.03% (lane b. ATF3) vs. 21.7 ± 2.08% (lane a. LacZ), **p < 0.01] was more significantly inhibited by pretreatment with an antibody specific to LIF than with control mouse IgG [attachment rate: 40.7 ± 12.01% (lane d. control IgG) vs. 26.0 ± 2.00% (lane c. LIF antibody), #*p* < 0.05; Fig. 4 F]. These results suggest that ATF3 regulates human endometrial receptivity and embryo attachment in vitro via up-regulation of leukemia inhibitory factor.

### Aberrantly low ATF3 expression in the endometria of RIF patients

**Fig. 5:**
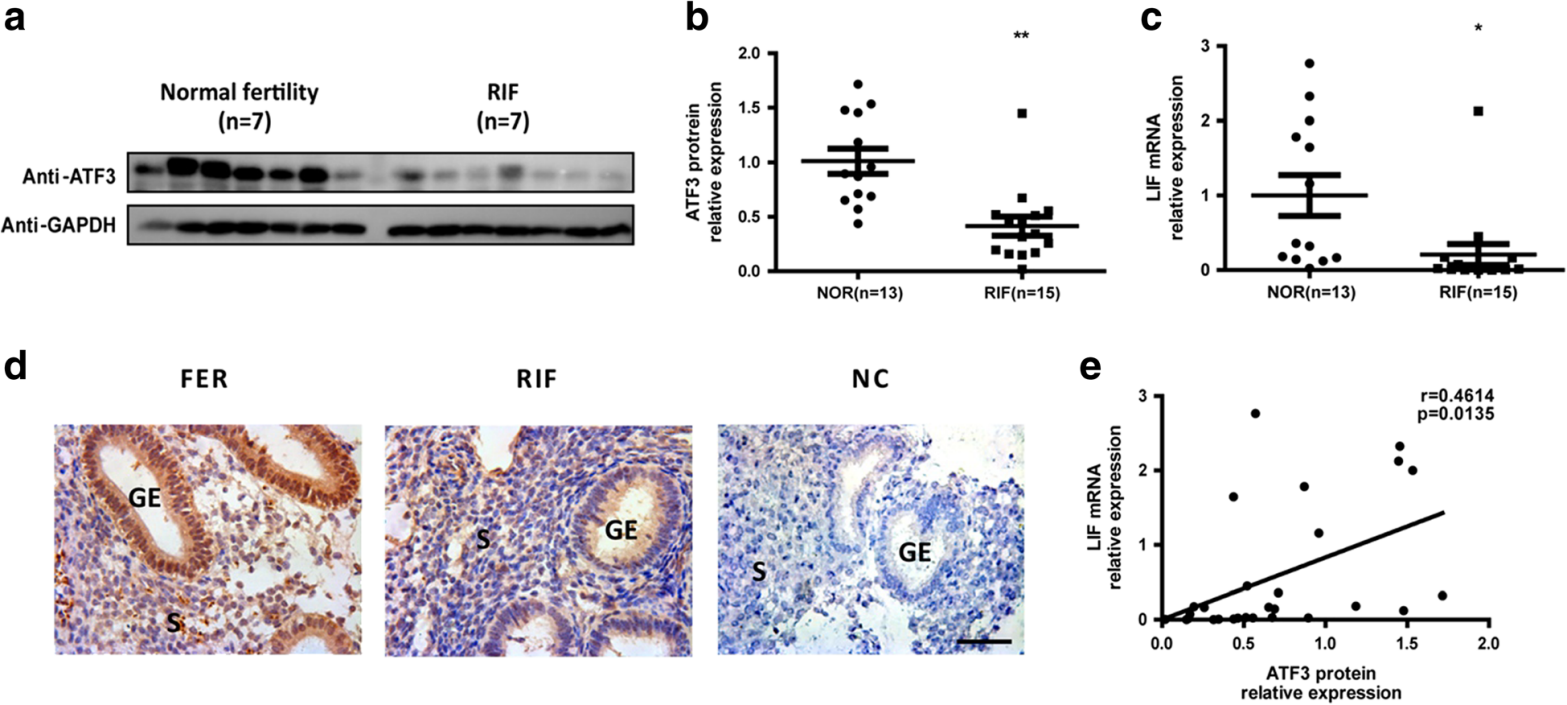
Aberrantly low ATF3 expression in the endometria of RIF patients. **a** Timed midsecretory endometrial biopsies from women with fertilized embryos (FER) (control, *n* = 7) and repeated implantation failure (RIF) patients (*n* = 7) were analyzed for ATF3 protein expression using Western blot analysis. Biopsies from FER women (control, *n* = 13) and RIF patients (*n* = 15) were analyzed for ATF3 protein expression by Western blot analysis (normalized to the GAPDH protein expression level) (**b**) and LIF mRNA level by real-time PCR (**c**). *p < 0.05; **p < 0.01 vs. the control group. **d** Secretory endometrial tissue samples from fertile control and RIF patients are shown at 400× magnification. The negative control (NC) is nonspecific rabbit serum. Brown staining represents positive staining (arrows). Scale bars, 50 μm. **e** Correlation between ATF3 protein expression and LIF mRNA expression in endometrial samples of FER women and RIF patients

It was previously reported that compared with control patients, RIF patients exhibit deregulated LIF expression during the receptive phase [23]. Here, we discovered a trend for reduced ATF3 protein expression (Fig. 5a and b) as well as reduced LIF mRNA content (Fig. 5c) in the endometria of RIF patients (*n* = 15) compared with that of fertile females who underwent embryo transplantation once (FER) (*n* = 13) (Table 1), and a positive correlation was established between ATF3 and LIF expression (*r* = 0.461, *p* = 0.013; Fig. 4E). The IHC results also demonstrated that ATF3 was aberrantly expressed at low levels in both the epithelial cells and the stromal cells of the RIF group (Fig. 4D), indicating that decreased ATF3 expression in the endometrium was associated with impaired endometrial receptivity.

## Discussion

As stress-inducible transcription factors, the ATF/CREB family members participate extensively in various essential physiological or pathological processes, such as cell cycling, immune functions and oncogenesis [12]. Mice homozygous for the ATF2 mutation showed decreased postnatal survival. In addition, ATF4 is required for the differentiation of the lamina propria layer of the vas deferens [24]. ATF3 is a potential target gene of estrogen in sexual differentiation [14], and it is involved in progesterone-related ovarian cancer [16] and luteal regression [25]. In this study, we demonstrate that ATF3 is responsive to steroid hormone induction in Ishikawa cells and promotes embryo adhesion in vitro by transcriptionally increasing LIF expression.

In humans, implantation is achieved only during a very short period in the midsecretory phase called the window of implantation, when complex interactions between multiple factors create a receptive-stage endometrium that is suitable for blastocyst implantation [1]. One of the secretions produced by the endometrial epithelium is the endometrial capacity marker LIF, which binds to the LIF receptor (LIFR) on the endometrial surface, recruits gp130 to form a high-affinity functional receptor complex, and contributes to the activation of the JAK-STAT pathway [6]. STAT3 phosphorylation is essential for the activity of LIF in regulating epithelial cell-cell junctions, polarity and functions during the receptive phase [7]. Moreover, LIF and LIFR are also located in endometrial stromal cells and play an important role in decidualization [9]. In mice, blocking the activity of LIF in the uterus using a PEGylated antagonist significantly blocked blastocyst implantation, and the embryos of LIF-null mice developed to the blastocyst stage but had difficulty during implantation [26].

In this study, we demonstrated that ATF3 was located in both human endometrial epithelial cells and stromal cells and was induced by steroid hormones in Ishikawa cells during the early stage. Overexpressing ATF3 in Ishikawa cells clearly up-regulated LIF expression, triggered STAT3 phosphorylation and consequently increased the ratio of BeWo spheroid adhesion. The reduction in ATF3-stimulated BeWo spheroid adhesion due to pretreatment with LIF-specific antibody and the positive correlation between ATF3 and LIF expression in endometrial samples indicate that ATF3 plays a significant role in endometrial capacity via the regulation of LIF expression during embryo implantation.

Previous studies have shown that LIF is adjusted by p53 [27], EPAC2 [28] and miR181a [29] in endometrial cells, which cannot fully explain its regulatory mechanism. Members of the ATF family bind to a consensus DNA sequence (TGACGTCA) and form selective heterodimers with each other via the leucine zipper region [17]. Here, we demonstrated that ATF3 transcriptionally up-regulated LIF by binding to its promoter region. Coincidentally, Yosef Buganimy [22] performed a chromatin immunoprecipitation assay with HK-3T^ATF3^ cells to test whether LIF is equipped with one or more ATF3/CREB-binding sites and can bind to the ATF3 promoter region. However, LIF mRNA levels were down-regulated by the expression of ATF3 in HK-3 T cells, which contrasts with our results. These different findings indicate that the transcriptional effect of ATF3 on LIF may be diverse in different cells and under different physiological or pathological conditions. Additional analyses are required to identify ATF3-specific binding sequences within the LIF, LIFR and gp130 promoter regions.

Endometrial decidualization involves the mesenchymal-to-epithelial transformation of endometrial stromal cells into secretory epithelioid decidual cells [1]. Impaired decidualization leads to undesirable stroma secretion, an unbalanced immune environment and disintegrating maternal-embryonic responses, predisposing patients to pregnancy loss [30]. Here, we discovered that ATF3 expression was abnormally reduced in both the endometrial epithelial cells and the stromal cells of RIF patients, indicating that ATF3 was involved in embryo adhesion and suggesting that ATF3 may be a favorable factor of decidualization. ATF3, recognized as a useful marker for the regenerative response of neuronal injury [31] and the migration of ependymal stem cells in the rat spinal cord [32], is a significant regulator of cell proliferation and differentiation. ATF3 also reportedly participates in Akt activation [33], the TGF-β pathway [34] and the transcriptional activity of foxo1 [35], which are all relevant to the decidualization process. Further studies should be conducted to determine whether ATF3 plays a role in the decidualization of stromal cells.

In summary, our study is the first to show that ATF3 is a novel downstream target gene of E_2_ and MPA in Ishikawa cells and facilitates embryo attachment in vitro by increasing LIF expression. A fresh understanding of how the endometrial receptivity marker gene LIF regulates this process will help to solve endometrial capacity-relevant infertilities in IVF programs, especially in patients with recurrent implantation failure or abortion.

## Conclusions

Together, this study highlights the relationship between ATF3 and LIF in regulating embryo attachment in vitro. Our findings provide novel potential biomarkers and targets for diseases associated with defective endometrial capacity.

## Additional files

Additional file 1: Table S1. Demographic details of the participants in the study of endometrial ATF3 expression of fertile control (FER) and RIF patients. **Table S2.** Demographic details of the participants in the study of endometrial ATF3 expression of proliferative and secretory phase endometria. (DOCX 18 kb)

Additional file 2: Figure S1. Antibodies against LIF or mouse preimmune IgG were cultured with treated cells at a concentration of 0, 0.1, 0.25, 0.5, 1, 2 μg/mL for 1 h before the transfer of the BeWo spheroids. **p <0.01 0.5 ng IgG vs. contorl. The error bars indicate ± SD of 3 independent experiments. **Figure S2.** Pre-treatment of ICI182780 and mifepristone were performed before sex hormones were perfomed with a time-dependent mammer. ATF3 mRNA expression was determined by qPCR. (DOCX 3222 kb)

Additional file 3: Editorial certification. (PDF 821 kb)

Additional file 4: The original IRB approval. (DOC 155 kb)

Additional file 5: English translation of the IRB approval. (DOC 64 kb)

## Abbreviations

ATF3: Activating transcription factor 3
ChIP: Chromatin immunoprecipitation
CREB: cAMP response element binding protein
LIF: Leukemia inhibitory factor
MOI: Multiplicity of infection
MPA: Medroxyprogesterone acetate
RIF: Recurrent implantation failure
STAT: Signal transducer and activator of transcription
