# Supplementary material for: Activating transcription factor 3 promotes embryo attachment via up-regulation of leukemia inhibitory factor in vitro
Source: Reprod Biol Endocrinol. 2017 Jun 2;15:42. doi: 10.1186/s12958-017-0260-7 (PMC5457579; doi:10.1186/s12958-017-0260-7)
Supplement: Supplementary file 1 — Demographic details of the participants in the study of endometrial ATF3 expression of fertile control (FER) and RIF patients. Table S2. Demographic details of the participants in the study of endometrial ATF3 expression of proliferative and secretory phase endometria. (DOCX 18 kb) [file 12958_2017_260_MOESM1_ESM.docx]

**Table 1** Demographic details of the participants in the study of endometrial ATF3 expression of fertile control (FER) and RIF patients

| **Fertile** | **FER(n=13)** | **RIF(n=15)** | **P** |
| --- | --- | --- | --- |
| **Age(years)** | 32.5±4.12 | 33.2±4.44 | 0.6877(ns) |
| **BMI(kg/m2)** | 20.7±2.11 | 21.5±2.28 | 0.385(ns) |
| **Endometrial thickness (mm)** | 9.1±1.50 | 10.6±2.24 | 0.125(ns) |
| **No. of transferred embryos** | 2±0 | 8.267±3.83 | 1.0533E-06(ns) |

**The data are presented as the mean ± SD unless otherwise indicated. A P < 0.05 was considered significant**

**Table 2** Demographic details of the participants in the study of endometrial ATF3 expression of proliferative and secretory phase endometria.

| **Phase of menstruation** | **Proliferative phase (n=10)** | **Secretory phase (n=10)** | **P** |
| --- | --- | --- | --- |
| **Age(years)** | 32.0± 3.80 | 34.6±3.03 | 0.108 (ns) |
| **BMI(kg/m2)** | 20.7±1.185 | 21.9±2.677 | 0.226(ns) |

**The data are presented as the mean ± SD unless otherwise indicated. A P < 0.05 was considered significant**
