# Supplementary material for: Activating transcription factor 3 promotes embryo attachment via up-regulation of leukemia inhibitory factor in vitro
Source: Reprod Biol Endocrinol. 2017 Jun 2;15:42. doi: 10.1186/s12958-017-0260-7 (PMC5457579; doi:10.1186/s12958-017-0260-7)
Supplement: Supplementary file 2 — Antibodies against LIF or mouse preimmune IgG were cultured with treated cells at a concentration of 0, 0.1, 0.25, 0.5, 1, 2 μg/mL for 1 h before the transfer of the BeWo spheroids. **p <0.01 0.5 ng IgG vs. contorl. The error bars indicate ± SD of 3 independent experiments. Figure S2. Pre-treatment of ICI182780 and mifepristone were performed before sex hormones were perfomed with a time-dependent mammer. ATF3 mRNA expression was determined by qPCR. (DOCX 3222 kb) [file 12958_2017_260_MOESM2_ESM.docx]

**
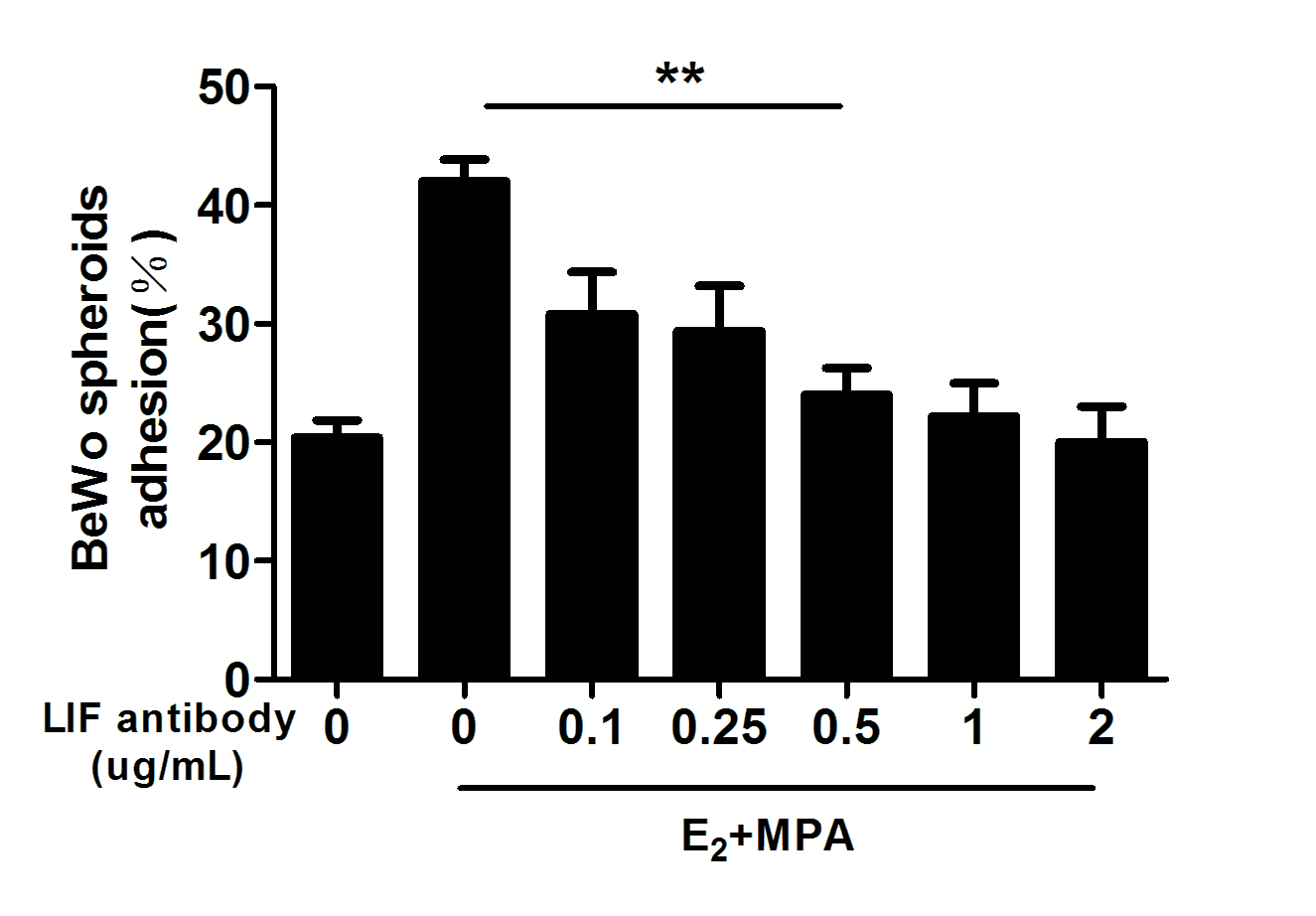
Fig. S1**

**Fig. S1 Antibodies against LIF or mouse preimmune IgG were cultured with treated cells at a concentration of 0, 0.1, 0.25, 0.5, 1, 2 μg/mL for 1 h before the transfer of the BeWo spheroids. **p <0.01 0.5 ng IgG *vs.* contorl. The error bars indicate ±SD of 3 independent experiments.**

**Fig. S2**

**
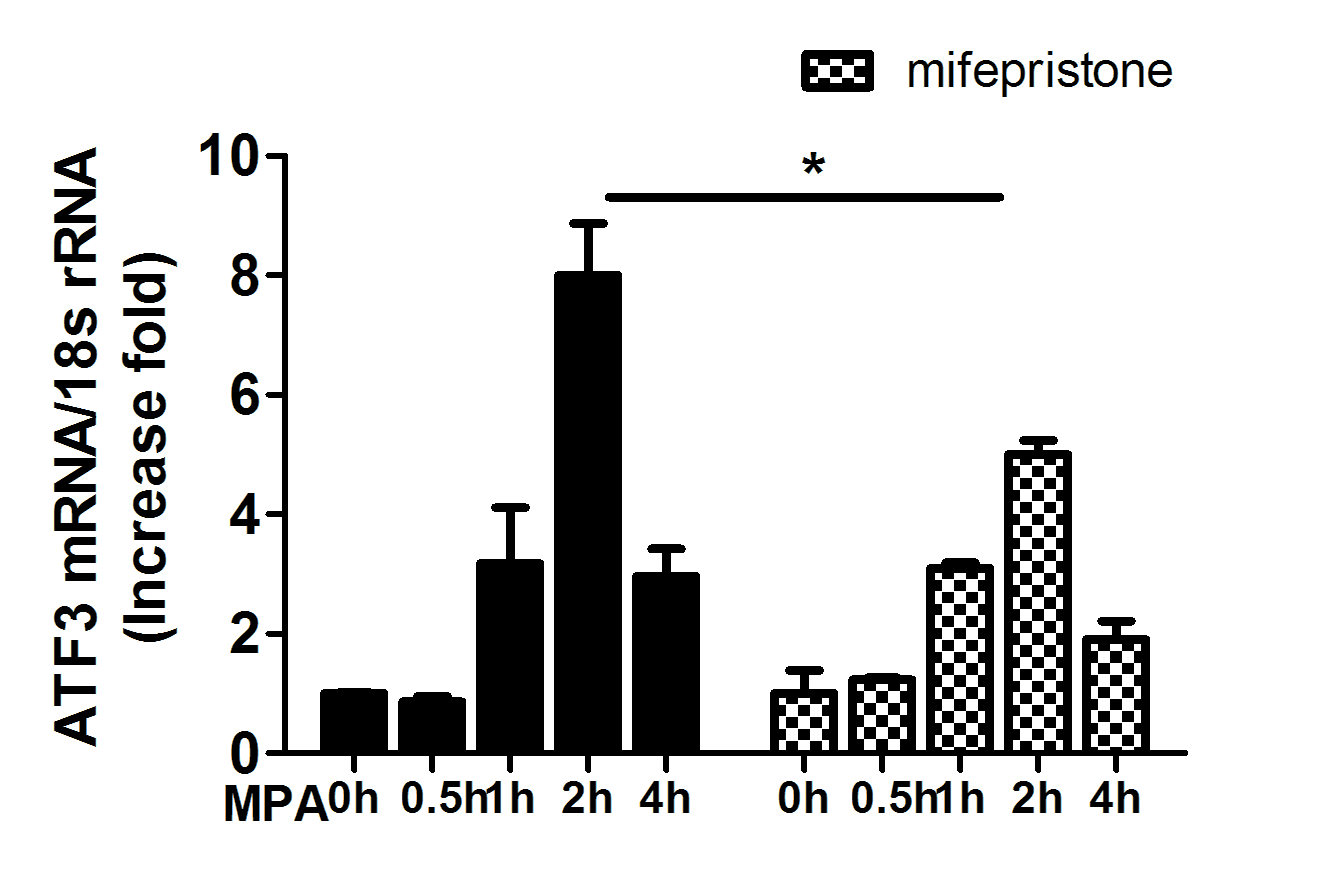

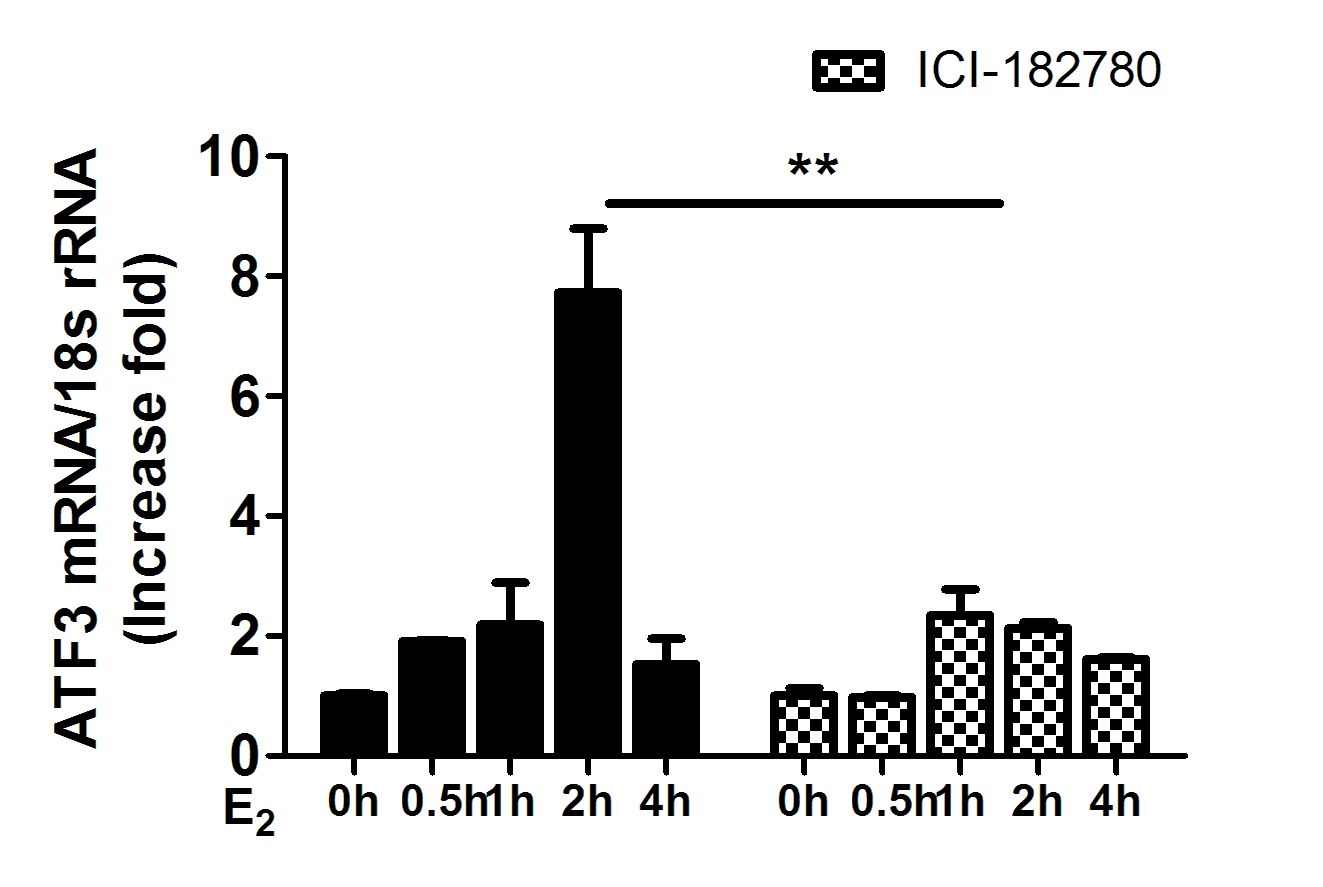

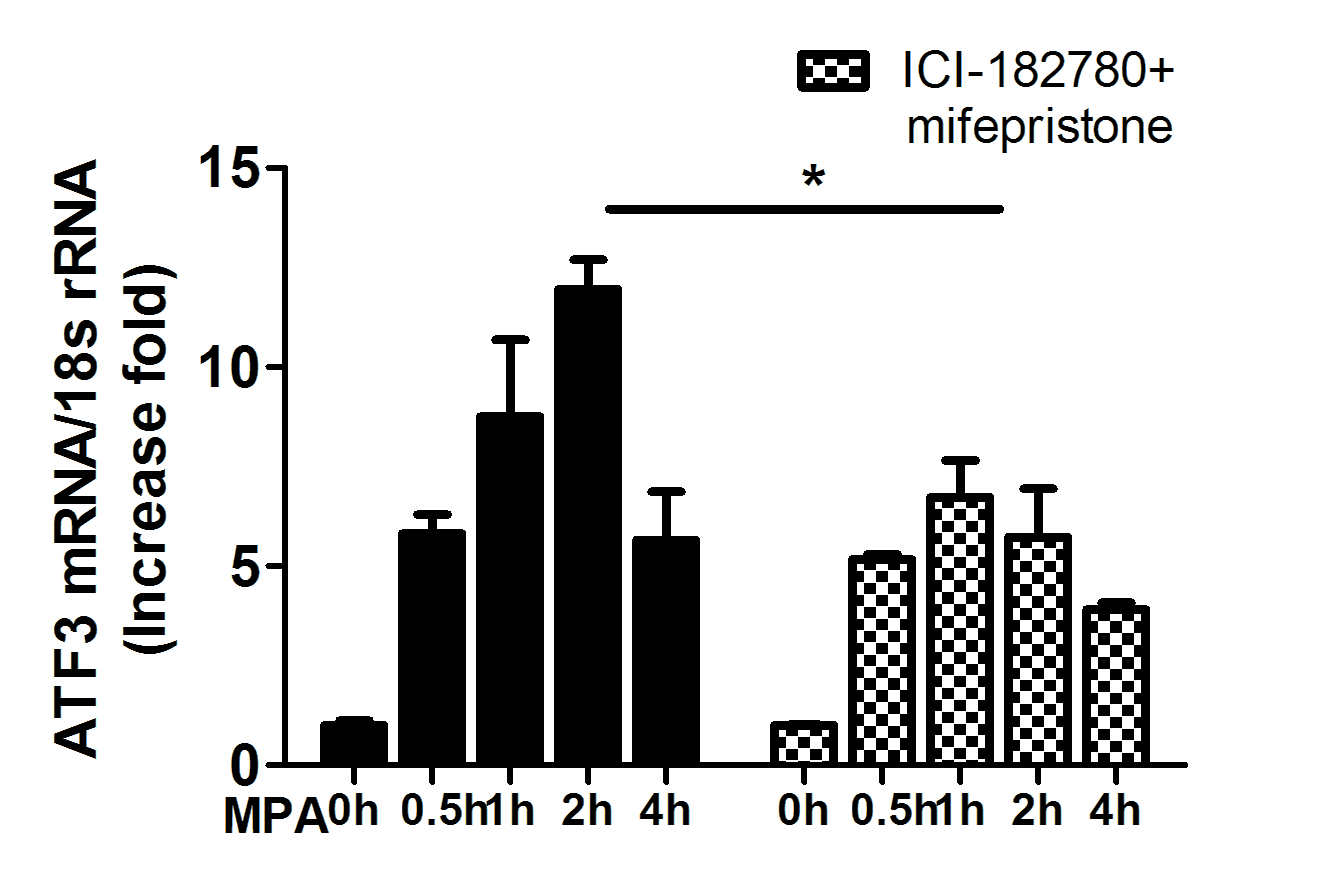
**

**Fig. S2 Pre-treatment of ICI182780 and mifepristone were performed before sex hormones were perfomed with a time-dependent mammer. ATF3 mRNA expression was determined by qPCR.**
