# Supplementary material for: Activating transcription factor 3 promotes embryo attachment via up-regulation of leukemia inhibitory factor in vitro
Source: Reprod Biol Endocrinol. 2017 Jun 2;15:42. doi: 10.1186/s12958-017-0260-7 (PMC5457579; doi:10.1186/s12958-017-0260-7)
Supplement: Supplementary file 3 — Editorial certification. (PDF 821 kb) [file 12958_2017_260_MOESM3_ESM.pdf]

AMERICAN JOURNAL EXPERTS

EDITORIAL CERTIFICATE

This document certifies that the manuscript listed below was edited for proper English language, grammar, punctuation, spelling, and overall style by one or more of the highly qualified native English speaking editors at American Journal Experts.

Manuscript title:

ATF3 promotes embryo adhesion in vitro though elevating LIF expression

Authors:

Xi Cheng, Jingyu Liu, Lihua Sun, Chenyang Huang, Qiang Yan, Jialei Shen, Ruiwei Jiang, Lijun Ding, Zhenyu Diao, Yue Jiang, Zhou Jianjun, Guijun Yan, Haixiang Sun

Date Issued:

March 23, 2016

Certificate Verification Key:

33BD-62F3-9A19-02E0-7E3C

This certificate may be verified at www.aje.com/certificate. This document certifies that the manuscript listed above was edited for proper English language, grammar, punctuation, spelling, and overall style by one or more of the highly qualified native English speaking editors at American Journal Experts. Neither the research content nor the authors' intentions were altered in any way during the editing process. Documents receiving this certification should be English-ready for publication; however, the author has the ability to accept or reject our suggestions and changes. To verify the final AJE edited version, please visit our verification page. If you have any questions or concerns about this edited document, please contact American Journal Experts at support@aje.com.

American Journal Experts provides a range of editing, translation and manuscript services for researchers and publishers around the world. Our top-quality PhD editors are all native English speakers from America's top universities. Our editors come from nearly every research field and possess the highest qualifications to edit research manuscripts written by non-native English speakers. For more information about our company, services and partner discounts, please visit www.aje.com.
